# Hepcidin, ferroportin, and hemoglobin as predictors of iron deficiency anemia risk and perinatal outcomes in twin pregnancy

**DOI:** 10.3389/fmed.2025.1703592

**Published:** 2025-11-24

**Authors:** Yanling Zhong, Lin Deng, Xingyan Xu, Jinfu Zhou, Jianying Yan, Jinying Luo

**Affiliations:** 1 Department of Gynaecology and Obstetrics, Fujian Maternity and Child Health Hospital, College of Clinical Medicine for Obstetrics & Gynecology and Pediatrics, Fujian Medical University, Fuzhou, China; 2 Department of Scientific Research, The Second People’s Hospital Affiliated to Fujian University of Chinese Medicine, Fuzhou, China; 3 Medical Genetic Diagnosis and Therapy Center, Fujian Maternity and Child Health Hospital, College of Clinical Medicine for Obstetrics & Gynecology and Pediatrics, Fujian Medical University, Fuzhou, China; 4 Fujian Clinical Research Center for Maternal-Fetal Medicine, Fuzhou, China; 5 Laboratory of Maternal-Fetal Medicine, Fujian Maternity and Child Health Hospital, Fuzhou, China

**Keywords:** twin pregnancy, iron deficiency anemia, hepcidin, ferroportin, perinatal outcomes

## Abstract

**Objective:**

This study investigated iron-deficiency anemia (IDA), third-trimester hepcidin and ferroportin levels, and perinatal outcomes in twin pregnancies.

**Study design:**

Pregnant women with twin gestations (*n* = 195) were enrolled and classified into an IDA group (*n* = 63; further subdivided into adverse and non-adverse perinatal-outcome subgroups) and a non–iron-deficiency anemia group (*n* = 132). Serum hepcidin and ferroportin were measured at ≥28 weeks of gestation. Analyses included multivariate logistic regression and receiver operating characteristic curve assessments.

**Results:**

The prevalence of IDA was 32.3%. Hepcidin showed positive correlations with red blood cell count, hemoglobin, hematocrit, mean corpuscular volume, mean corpuscular hemoglobin, and mean corpuscular hemoglobin concentration. Ferroportin showed negative correlations with these indices as well as with hepcidin. Adverse outcomes occurred in 84.1% of pregnancies complicated by iron-deficiency anemia. The combined decrease in hepcidin and hemoglobin levels, together with an increase in ferroportin concentrations, yielded an area under the curve of 0.940 for predicting adverse outcomes.

**Conclusion:**

IDA is common in twin pregnancies and is strongly associated with unfavorable maternal–fetal outcomes. The combined measurement of hepcidin, ferroportin, and hemoglobin has diagnostic and predictive value in assessing iron-deficiency anemia in twin pregnancies.

## 1 Introduction

Anemia is a common public health concern in obstetrics, affecting more than 36% of pregnant women worldwide (1). It also can lead to various complications during pregnancy (2, 3). It can result from nutritional deficiencies, thalassemia, or megaloblastic anemia. The most frequent form of nutritional-deficiency anemia arises from malnutrition, leading to inadequate intake of nutrients such as iron, folic acid, vitamin B12, vitamin B6, vitamin A, vitamin C, and protein, all of which are essential for hemoglobin (HGB) synthesis and red blood cell (RBC) production. Iron deficiency is the predominant cause, accounting for over 50% of anemia cases and giving rise to iron-deficiency anemia (IDA) (4). Currently, no universally accepted diagnostic standard for IDA exists. However, the most recent Chinese guidelines recommend diagnostic thresholds of HGB < 110 g/L, transferrin saturation (TSAT) < 15%, and serum ferritin (SF) < 20 μg/L (5). Additionally, the World Health Organization defines maternal anemia as HGB < 110 g/L and further classifies severity as mild (100–109 g/L), moderate (70–99 g/L), or severe (< 70 g/L). In clinical practice, combining a full blood cell (FBC) count with SF and vitamin B12 testing is essential for differentiating IDA from other types of anemia during pregnancy (6).

A twin pregnancy is defined as the simultaneous presence of two fetuses in the uterus. Its incidence has risen with changes in national fertility policies and the increasing use of assisted reproductive technologies. Twin pregnancy is recognized as a high-risk condition associated with adverse perinatal outcomes such as preterm birth, postpartum hemorrhage, placental abruption, gestational hypertension, fetal growth restriction, and neonatal asphyxia (7). The co-occurrence of IDA further amplifies these risks, and the incidence of IDA in twin pregnancies is estimated to be 2.4 to 4 times higher than in singleton pregnancies. This increased susceptibility is explained by the greater expansion of maternal blood volume and the higher nutritional demands of two fetuses compared with singleton pregnancies (8, 9).

Hepcidin (Hepc), also known as a liver-derived antimicrobial peptide, is a cysteine-rich peptide hormone that is secreted into the blood and excreted in the urine. Hepc exerts its function by regulating ferroportin (FPN), the only identified iron-export protein located on the cell membrane. In mice, FPN knockout results in embryonic lethality due to severe iron deficiency (10). FPN is highly expressed in duodenal epithelial cells, liver and spleen macrophages, and placental trophoblasts, where it mediates the export of iron from cells into the plasma. Binding of Hepc to FPN induces its degradation, thereby reducing iron release into the plasma and decreasing systemic iron bioavailability (11). Hepc synthesis is regulated by iron levels, erythropoietic activity, and inflammation (12). During iron deficiency, anemia, or hypoxia, Hepc production is suppressed, leading to enhanced intestinal iron absorption and mobilization of stored iron from macrophages and hepatocytes. Conversely, Hepc production increases in response to iron overload, inflammation, or infection, which downregulates FPN expression. Hepc is therefore a central regulator of iron metabolism, showing positive correlations with HGB levels and negative correlations with FPN expression (13). The discovery of the Hepc–FPN regulatory pathway has provided new perspectives for the diagnosis and treatment of IDA during pregnancy (14, 15); however, its role in the pathophysiology of twin pregnancy remains unclear.

Given the high-risk nature of twin pregnancies, this study aimed to investigate the effects of IDA on maternal and perinatal outcomes and to examine the relationship between serum Hepc and FPN levels and IDA, as well as their impact on perinatal outcomes. A further objective was to provide a clinical reference for the diagnosis and management of IDA in twin pregnancies.

## 2 Materials and methods

### 2.1 Patients

**Figure 1:**
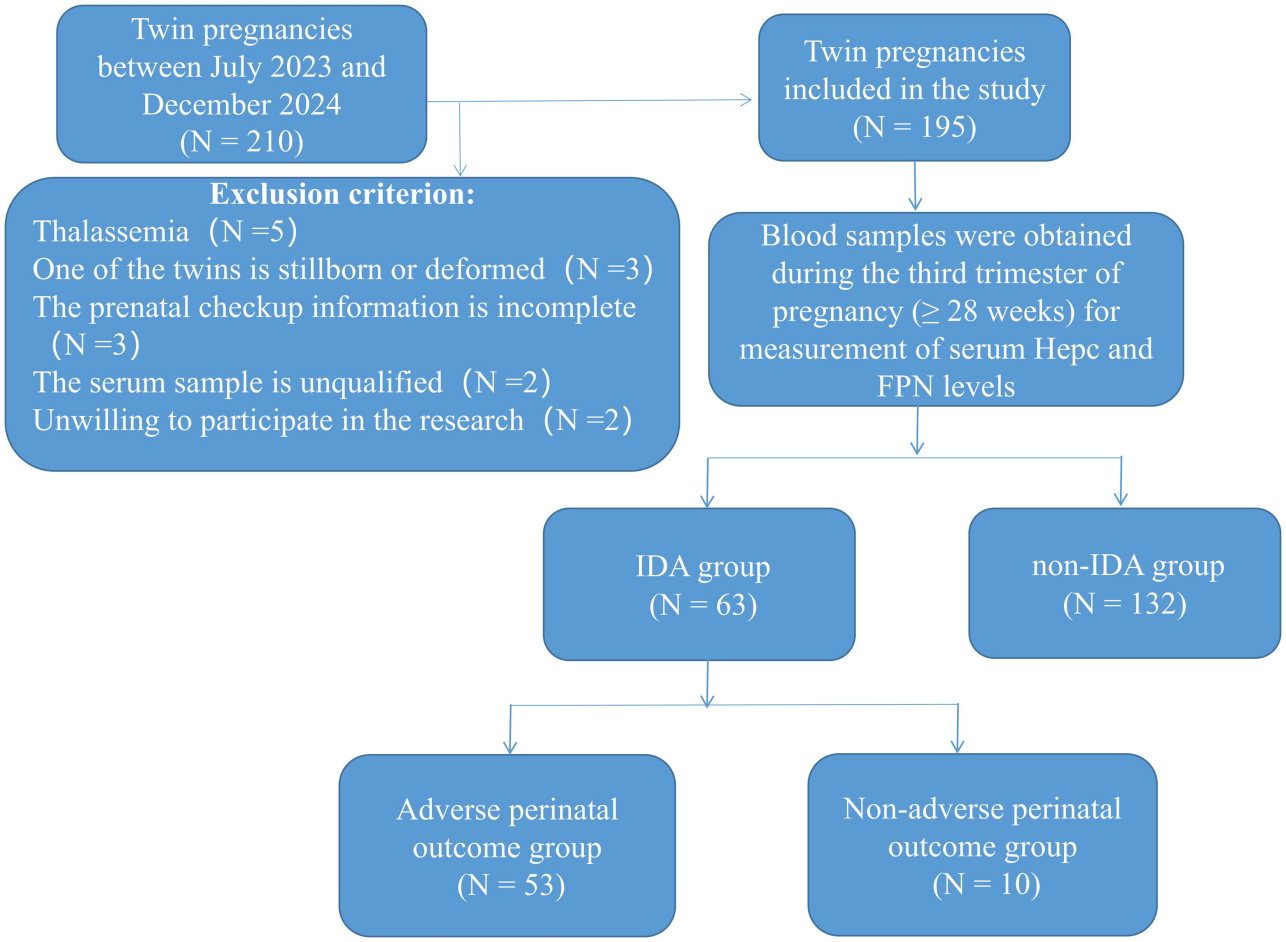
The participant flow diagram.

A total of 195 women with twin pregnancies, aged 18–45 years, who registered and delivered at Fujian Maternity and Child Health Hospital between July 2023 and December 2024 were enrolled. The participant flow diagram is shown in Figure 1. Blood samples were obtained during the third trimester of pregnancy (≥ 28 weeks) for measurement of serum Hepc and FPN levels. Hepc and FPN concentrations were determined using enzyme-linked immunosorbent assay (ELISA). Written informed consent was obtained from the participants or their legal guardians, including consent for the publication of any potentially identifiable images or data included in this study. The study protocol was approved by the Medical Ethics Committee of Fujian Maternity and Child Health Hospital (Approval Number: 2023KY136).

### 2.2 Clinical data collection

General patient information, including age, chorionicity, mode of conception, pre-pregnancy body mass index (BMI), obstetric history, and medical history, was obtained from the electronic medical record system. Pregnancy-related indicators such as blood pressure, weight, and auxiliary examination results (routine blood tests and ultrasound) were monitored and documented during prenatal visits. Maternal outcomes, including placental size and weight, umbilical cord length and insertion site, and amniotic fluid characteristics, were recorded, as were neonatal outcomes such as gestational age at delivery, sex, birth weight, and Apgar score. FBC indices included white blood cell count, neutrophil count, lymphocyte count, RBC count, HGB, hematocrit (HCT), mean corpuscular volume (MCV), mean corpuscular hemoglobin (MCH), mean corpuscular hemoglobin concentration (MCHC), red cell distribution width–coefficient of variation, red cell distribution width–standard deviation, plateletcrit (PCT), and mean platelet volume (MPV).

All women with twin pregnancies were followed until 3 days postpartum. The primary outcomes included preterm birth, preeclampsia (PE), placental adhesion, placental abruption, premature rupture of membranes, umbilical cord torsion, nuchal cord, meconium-stained amniotic fluid, postpartum hemorrhage, neonatal asphyxia in either twin, and small-for-gestational-age in either twin.

### 2.3 Evaluation criteria

Twin pregnancies were identified by early pregnancy ultrasound at 8–10 weeks or by nuchal translucency (NT) color Doppler ultrasound at 11–13 + 6 weeks and were confirmed postpartum. IDA was diagnosed based on clinical manifestations and third-trimester peripheral blood testing. Mild IDA was characterized by pale skin and mucous membranes with minimal or no symptoms, whereas severe IDA could present with pallor, fatigue, dizziness, tinnitus, blurred vision, palpitations, dyspnea, fainting, hypoalbuminemia, edema, or ascites. The diagnostic criteria for IDA were HGB < 110 g/L and SF < 20 μg/L. The non-IDA group consisted exclusively of women without IDA or other forms of anemia (e.g., thalassemia, B12 deficiency).

### 2.4 Blood collection and Indicator measurement

Venous blood samples (5–8 mL) were collected between 8:00 and 10:00 after an overnight fast of at least 8 h. Blood was drawn into coagulation tubes and centrifuged at 3,000 rpm/min for 10 min. The resulting supernatant (3 mL) was aliquoted into cryovials, labeled, and immediately stored at −80 °C. Prior to testing, samples were thawed at 18–20 °C for 1 h, centrifuged at 1,000 rpm/min for 20 min, and the supernatant was used for analysis.

Serum Hepc and FPN concentrations were determined by ELISA. High-sensitivity ELISA kits (Hangzhou Bosimeier Biotechnology Co., Ltd., China) were used in duplicate to measure Hepc and FPN levels (ng/mL). Assays were performed in batches with a SpectraMax 190 microplate reader (Molecular Devices, San Jose, CA, USA). Based on the principle of competitive binding, the intensity of the colorimetric reaction was directly proportional to the Hepc or FPN concentration in patient samples.

### 2.5 Statistical analysis

Data were entered into a database using Microsoft Excel and analyzed with SPSS version 27.0 (IBM Corp., Armonk, NY, USA). Normally distributed continuous variables are presented as mean ± standard deviation (x̄ ± s), and between-group comparisons were performed using independent-samples t-tests. Categorical variables are expressed as frequencies and percentages and were analyzed with chi-square tests or Fisher’s exact test, as appropriate. Univariate and multivariate logistic regression models were applied to identify factors associated with IDA in twin pregnancies and to assess their relationship with perinatal outcomes. A two-sided *p* value < 0.05 was considered statistically significant.

## 3 Results

### 3.1 Comparison of baseline characteristics between twin pregnancy groups

Among the 195 cases, the number of monozygotic and dizygotic twins was 63 and 132, respectively. Blood samples were collected at 32.13 ± 2.78 weeks’ gestation. The incidence of IDA in the third trimester of twin pregnancies was 32.31% (63/195). There were no significant differences between the IDA and non-IDA groups in age, gestational weight gain, gestational age at enrollment, pre-pregnancy BMI, chorionicity, mode of conception, C-section history, or miscarriage history (*p* > 0.05) (Table 1).

**Table 1:** Comparison of baseline characteristics between the IDA and non-IDA groups.

Variables	IDA group (*N* = 63) Mean ± SD	Non-IDA group (*N* = 132) Mean ± SD	*t or χ^2^*	*p*
Age (years)	30.33 ± 4.15	31.48 ± 3.90	1.888	0.060
Gestational weight gain (kg)	15.84 ± 10.19	15.83 ± 5.70	−0.013	0.990
Gestational age at enrollment (weeks)	10.08 ± 1.70	10.68 ± 1.76	1.758	0.081
Pre-pregnancy BMI (kg/m^2^)	21.64 ± 3.24	21.75 ± 2.93	0.299	0.819
Chorionicity (MCT)	23 (36.51%)	41 (31.06%)	0.574	0.447
Mode of conception (IVF-ET)	25 (39.68%)	65 (49.24%)	1.568	0.210
C-section history	9 (14.29%)	11 (8.33%)	1.642	0.200
Miscarriage history	4 (6.35%)	3 (2.27%)	2.048	0.152

IDA, iron deficiency anemia; BMI, body mass index; MCT, monochorionic twins; IVF-ET, in vitro fertilization and embryo transfer technology.

### 3.2 Differences in Hepc and FPN levels between twin pregnancy groups

Serum Hepc levels were significantly lower in the IDA group compared with the non-IDA group, whereas FPN levels were significantly higher in the IDA group (*p* < 0.05) (Table 2).

**Table 2:** Comparison of Hepc and FPN between the IDA and non-IDA groups.

Variables	IDA group (*N* = 63) Mean ± SD	Non-IDA group (*N* = 132) Mean ± SD	*t*	*p*
Hepc (ng/mL)	7.39 ± 7.17	12.99 ± 9.50	4.155	<0.001
FPN (ng/mL)	18.70 ± 10.14	14.39 ± 9.37	−2.921	0.004

IDA, iron deficiency anemia; Hepc, hepcidin; FPN, ferroportin.

### 3.3 Correlation analysis of Hepc, FPN, and hematologic indices

**Figure 2:**
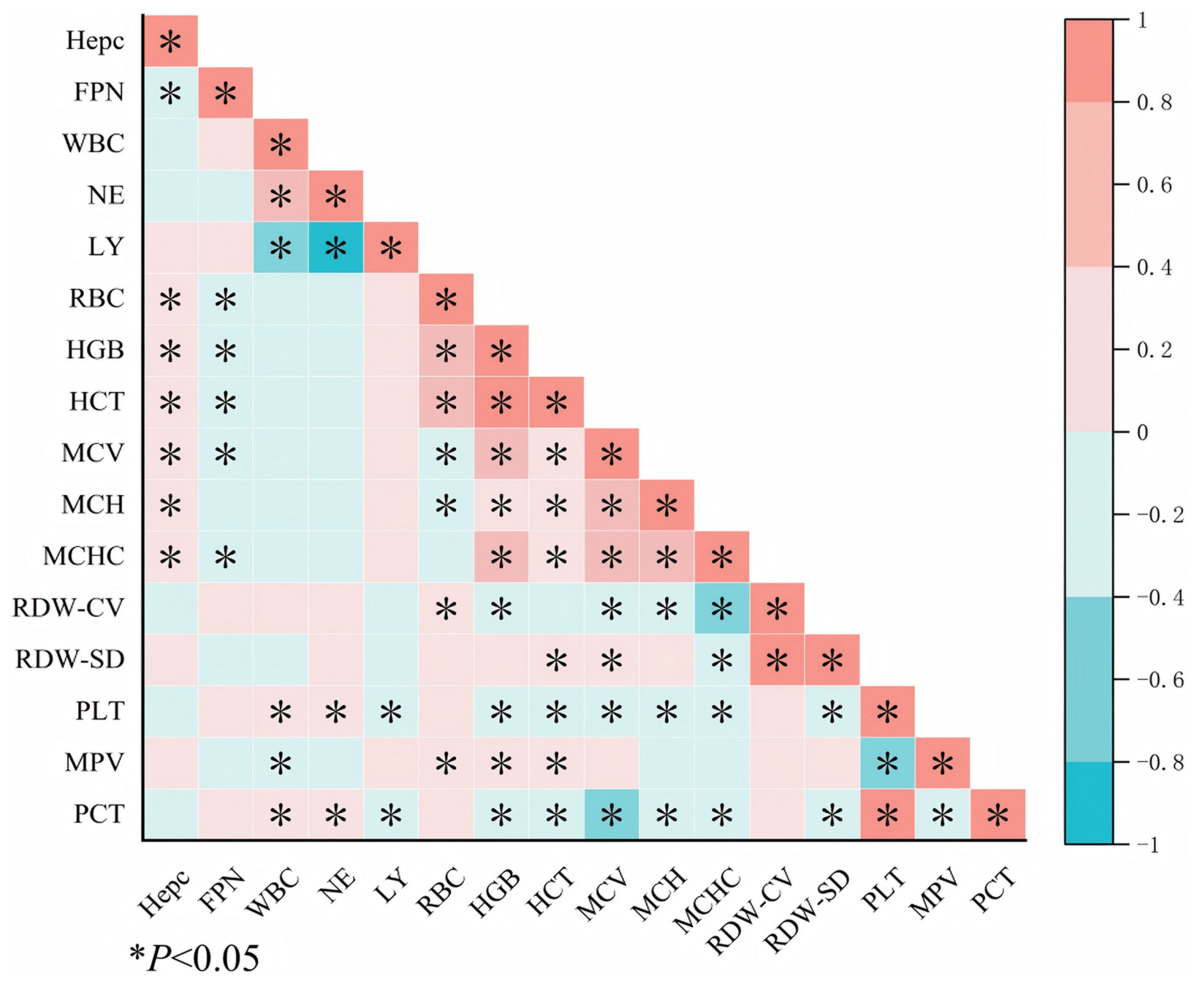
Correlation analysis of Hepc, FPN, and blood routine indicators. Red represents positive correlation, and blue represents negative correlation. Hepc, hepcidin; FPN, ferroportin; HGB, hemoglobin; WBC, white blood cell; NE, neutrophilic granulocyte; LY, lymphocyte; RBC, red blood cell; HCT, hematocrit; MCV, mean corpuscular volume; MCH, mean corpuscular hemoglobin; MCHC, mean corpuscular hemoglobin concentration; PLT, platelet; MPV, mean platelet volume; PCT, platelet hematocrit; RDW-CV, red cell distribution width coefficient of variation; RDV-SD, red cell distribution width-standard deviation.

Serum Hepc levels were positively correlated with RBC count, HGB, HCT, MCV, MCH, and MCHC (*p* < 0.05). In contrast, FPN levels were negatively correlated with Hepc, RBC count, HGB, HCT, MCV, and MCHC (*p* < 0.05) (Figure 2).

### 3.4 Relationship between Hepc and adverse perinatal outcomes of IDA in twin pregnancies

#### 3.4.1 Perinatal outcomes in the IDA group

Based on the presence or absence of concurrent IDA, patients were classified into an IDA group (*n* = 63) or a non-IDA group (*n* = 132). Among the 63 women with IDA, all the patients delivered via scheduled or emergency C-section (50 vs. 13 cases), and were further stratified into an adverse perinatal outcome group (*n* = 53) and a non-adverse perinatal outcome group (n = 10) according to the occurrence of adverse outcomes. Fifty-three (84.13%) women with IDA experienced at least one adverse perinatal outcome. These included 43 cases of preterm birth (68.25%), 14 cases of premature rupture of membranes (22.22%), 11 cases of PE (17.46%), 8 cases of small-for-gestational-age neonates, 6 cases of nuchal cord, 4 cases of placental adhesion, 3 cases of neonatal asphyxia, 2 cases of postpartum hemorrhage, 2 cases of placental abruption, 2 cases of meconium-stained amniotic fluid, and 1 case of umbilical cord torsion.

Considering IDA occurrence in the third trimester of pregnancy as the dependent variable (yes = 1, no = 0) and the various adverse perinatal outcomes observed as independent variables, binary logistic regression analysis was conducted. The results showed that the IDA group was more prone to preterm birth (OR = 2.271, 95% CI: 1.541–3.347, *p* < 0.01) and PE (OR = 1.795, 95% CI: 1.046–3.081, *p* < 0.05) compared to the non-IDA group. After adjusting for the influence of factors such as perinatal and chorionic factors, the results still showed statistically significant differences in the incidence of preterm birth and preeclampsia between the two groups. The risk of preterm birth in the IDA group was 2.487 times that in the non-IDA group (OR = 2.487, 95% CI: 1.633–3.787, *p* < 0.01), while the risk of preeclampsia was 1.954 times that in the non-IDA group (OR = 1.954, 95% CI: 1.108–3.446, *p* < 0.05).

#### 3.4.2 Comparison of clinical characteristics of different pregnancy outcomes

Hepc, HGB, and HCT levels were significantly lower in the adverse-outcome group than in the non-adverse-outcome group, whereas FPN levels were significantly higher (*p* < 0.05). No significant differences were observed between the two groups in age, pre-pregnancy BMI, gestational weight gain, gestational age at enrollment, or other anemia indices (*p* > 0.05) (Table 3).

**Table 3:** Comparison of clinical characteristics and indices related to iron metabolism of different perinatal outcomes in the IDA group.

Variables	Adverse perinatal outcome group (*N* = 53) Mean ± SD	Non-adverse perinatal outcome group (*N* = 10) Mean ± SD	*t*	*p*
Age (years)	30.38 ± 4.40	30.10 ± 2.60	−0.192	0.848
Gestational weight gain (kg)	21.55 ± 3.32	22.11 ± 2.89	0.497	0.621
Gestational age at enrollment (weeks)	16.02 ± 10.78	14.90 ± 6.67	−0.316	0.753
Pre-pregnancy BMI (kg/m^2^)	9.97 ± 1.74	10.57 ± 1.51	0.846	0.403
Hepc (ng/mL)	5.88 ± 5.83	15.38 ± 8.57	3.362	0.007
FPN (ng/mL)	20.15 ± 10.31	11.03 ± 3.97	−4.820	<0.001
HGB (g/L)	99.47 ± 6.60	104.70 ± 3.83	2.419	0.019
WBC (10^9^/L)	8.78 ± 2.49	10.37 ± 3.89	1.238	0.243
NE (%)	72.96 ± 7.31	73.90 ± 6.77	0.378	0.707
LY (%)	19.81 ± 6.68	18.99 ± 6.29	−0.359	0.721
RBC (10^12^/L)	3.52 ± 0.36	3.71 ± 0.23	1.593	0.116
HCT (%)	30.77 ± 1.87	31.62 ± 0.86	2.255	0.032
MCV (fl)	87.85 ± 7.66	88.36 ± 5.99	0.201	0.842
MCH (pg)	28.66 ± 3.39	28.63 ± 2.63	−0.025	0.980
MCHC (g/L)	325.32 ± 13.25	323.4 ± 11.32	−0.429	0.669
PLT (10^9^/L)	212.3 ± 55.06	232.6 ± 50.37	1.082	0.283
MPV (fl)	10.32 ± 0.97	10.24 ± 0.85	−0.231	0.818
PCT (%)	0.22 ± 0.05	0.24 ± 0.05	1.036	0.304

IDA, iron deficiency anemia; Hepc, hepcidin; FPN, ferroportin; HGB, hemoglobin; WBC, white blood cell; NE, neutrophilic granulocyte; LY, lymphocyte; RBC, red blood cell; HCT, hematocrit; MCV, mean corpuscular volume; MCH, mean corpuscular hemoglobin; MCHC, mean corpuscular hemoglobin concentration; PLT, platelet; MPV, mean platelet volume; PCT, platelet hematocrit.

#### 3.4.3 Multivariate analysis of adverse perinatal outcomes in twin pregnancies complicated with IDA

Variables identified as significant in the univariate analysis were entered into a multivariate logistic regression model, with adverse perinatal outcome (Yes = 1, No = 0) as the dependent variable. Decreased HGB, decreased Hepc, and increased FPN were independent risk factors for adverse perinatal outcomes (*p* < 0.05) (Table 4).

**Table 4:** Multivariate analysis of adverse perinatal outcomes in twin pregnancies complicated with IDA.

Variables	*β*	S. E.	Wald	*p*	OR	95% CI
HGB	−0.296	0.147	4.036	0.045	0.744	0.557–0.993
Hepc	−0.295	0.136	4.733	0.030	0.744	0.571–0.971
FPN	0.311	0.136	5.222	0.022	1.365	1.045–1.782
HCT	0.179	0.640	0.078	0.780	1.196	0.341–4.193

IDA, iron deficiency anemia; HGB, hemoglobin; Hepc, hepcidin; FPN, ferroportin; HCT, hematocrit.

#### 3.4.4 Predictive value of Hepc, FPN, and HGB for adverse perinatal outcomes in patients with IDA in twin pregnancies

**Figure 3:**
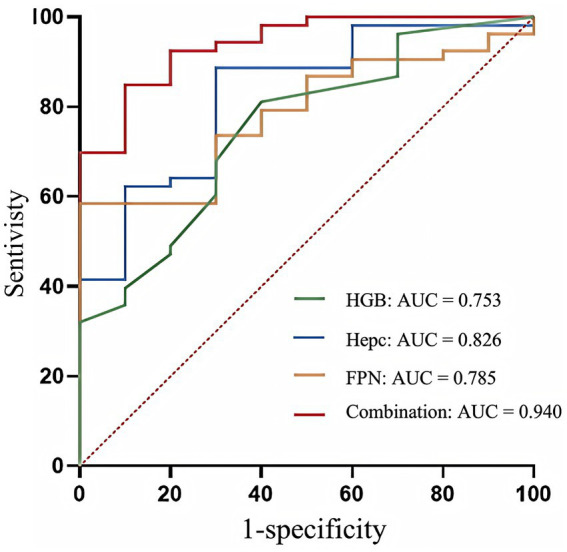
The ROC curves of Hepc, FPN and HGB for predicting adverse perinatal outcomes of IDA in twin pregnancies. The ROC curve evaluates the performance of classification models by mapping the relationship between the false positive rate (1-specificity) and the true positive rate (sensitivity). False positive rate (1-specificity), indicating the proportion of samples that were wrongly predicted to be positive among all those that were actually negative. IDA, iron deficiency anemia; HGB, hemoglobin; Hepc, hepcidin; FPN, ferroportin; AUC, area under the curve; ROC, receiver operating characteristic.

Receiver operating characteristic (ROC) curve analysis was performed to evaluate the predictive value of Hepc, FPN, and HGB for adverse outcomes in women with IDA twin pregnancies. The area under the curve (AUC) of Hepc was 0.826 (95% confidence interval [CI]: 0.695–0.958), the optimal cutoff value was 11.83 ng/mL, and the sensitivity, specificity, and maximum Youden index were 0.887, 0.700, and 0.587, respectively. The AUC of FPN was 0.785 (95% CI: 0.663–0.907), the optimal cutoff value was 15.96 ng/mL, and the sensitivity, specificity, and maximum Youden index were 0.585, 1.000, and 0.585, respectively. The AUC of HGB was 0.753 (95% CI: 0.597–0.909), the optimal cutoff value was 104.50 g/L, and the sensitivity, specificity, and maximum Youden index were 0.811, 0.600, and 0.411, respectively. The AUC of the combined diagnosis of the three was 0.940 (95% CI: 0.872–1.000), the optimal cutoff value was 0.85 ng/mL, and the sensitivity, specificity, and maximum Youden index were 0.849, 0.900, and 0.749, respectively. These findings demonstrate that Hepc, FPN, and HGB each have predictive value for adverse perinatal outcomes in IDA twin pregnancies, and that the combined model provides superior predictive performance (Table 5; Figure 3).

**Table 5:** ROC curve analysis of HGB, Hepc, and FPN for predicting adverse perinatal outcomes in twin pregnancies complicated with IDA.

Variables	AUC (95% CI)	Cut-off	Sensitivity	Specificity	Maximum Youden’s index
HGB (g/L)	0.753 (0.597–0.909)	104.50	0.811	0.600	0.411
Hepc (ng/mL)	0.826 (0.695–0.958)	11.83	0.887	0.700	0.587
FPN (ng/mL)	0.785 (0.663–0.907)	15.96	0.585	1.000	0.585
Combination	0.940 (0.872–1.000)	0.85	0.849	0.900	0.749

ROC, receiver operating characteristic; IDA, iron deficiency anemia; AUC, area under the curve; HGB, hemoglobin; Hepc, hepcidin; FPN, ferroportin; HCT, hematocrit.

## 4 Discussion

In this study, the incidence of IDA in the third trimester of twin pregnancies was 32.31%. Hepc levels were lower in the IDA group than in the non-IDA group, whereas FPN levels were higher. Serum Hepc was positively correlated with RBC count, HGB, HCT, MCV, MCH, and MCHC, while FPN was negatively correlated with these indices and with Hepc. Among women with IDA, 84.13% experienced adverse perinatal outcomes, most commonly preterm birth and PE. Hepc, HGB, and HCT levels were significantly lower in the adverse-outcome group compared with the non-adverse-outcome group, whereas FPN levels were higher. Low HGB and Hepc levels and elevated FPN were independent risk factors for adverse perinatal outcomes in twin pregnancies with IDA. Hepc, FPN, and HGB each demonstrated predictive value, and their combined use provided superior predictive performance.

A large domestic multicenter study reported that the incidence of anemia in twin pregnancies increases with gestational age, reaching 29.2% in the third trimester (16), which is consistent with the findings of our study. The demand for iron rises sharply during pregnancy because of hemodilution and the nutritional requirements of the fetus. In twin pregnancies, iron requirements increase substantially; however, maternal iron intake and absorption often fail to meet these demands, particularly in multiparous women (8, 17, 18). Maintenance of iron homeostasis therefore relies largely on maternal iron stores.

Several additional factors may influence the development of IDA in women with twin pregnancies. Low socioeconomic and educational status may limit access to nutritional and medical support, thereby increasing the risk of IDA (19, 20). Furthermore, maternal age at the extremes (advanced or very young), low pre-pregnancy BMI, a history of multiple pregnancies, and previous miscarriages have all been associated with an increased risk of IDA (19, 21). These observations underscore the need for medical staff to obtain thorough medical histories and to strengthen the management of women with twin pregnancies who are exposed to these risks in order to prevent and treat IDA effectively.

With respect to perinatal outcomes, the incidence of preterm birth among women with twin pregnancies complicated by IDA was 51%. Preterm births may be either spontaneous or iatrogenic. Iatrogenic preterm birth frequently occurs in twin pregnancies complicated by concurrent conditions, and nearly one-third of preterm births in twin pregnancies fall into this category (22). In such situations, pregnancy termination may be necessary. However, current domestic and international guidelines do not provide clear recommendations regarding the timing or mode of delivery for twin pregnancies complicated by IDA, and anemia itself is not considered an indication for iatrogenic preterm delivery. In the United States, 57% of twins are delivered by C-section. In many cases, even if the first baby is delivered vaginally, the birth of the second sibling may be performed by C-section, and the mean gestational age of twins at delivery is 35 weeks (23). In China, it is recommended that delivery can be considered at 38 weeks for dichorionic twins without complications or comorbidities. Monochorionic diamniotic (MCDA) twins without complications or comorbidities can be delivered under close monitoring after 37 weeks. For monochorionic and monoamniotic (MCMA) twins, delivery is recommended between 32 and 34 weeks. Complex twins (such as TTTS, sIUGR, and anemia-polyblood sequence in twins, etc.) require individualized delivery plans to be formulated based on the specific conditions of each pregnant woman and fetus. A trial of vaginal delivery can be chosen for MCDA twins and DCDA twins without complications. For MCMA twins, cesarean section is recommended (24). In this study, the preterm birth rate in the IDA group was significantly higher than that in the non-IDA group (68.25% vs. 47.73%, *p*<0.05), indicating that IDA contributes to the increased risk of preterm delivery in twin pregnancies. Physiological changes such as increased placental mass, reduced maternal antibody levels, and altered hormone secretion in twin pregnancies may also promote uterine contractions and elevate preterm birth risk (25–28). In addition, risk factors such as a history of miscarriage, assisted reproduction, PE, placenta previa, and cervical insufficiency are known to predispose to preterm birth in twin pregnancies (29–31). These variables were therefore adjusted for in our analysis; binary logistic regression analysis was conducted, and the results are described in Section 3.4.1: Perinatal Outcomes in the IDA group.

PE is a serious obstetric complication that can lead to liver and kidney damage, placental abruption, disseminated intravascular coagulation, fetal growth restriction, neonatal asphyxia, and even maternal or neonatal death. In this study, twin pregnancies complicated by IDA were closely associated with PE, with an incidence of 10.8%. In China, the incidence of PE is approximately 4.5% (32). Twin pregnancy is an independent risk factor for PE, with a rate 2–4 times higher than that of singleton pregnancies (33). Because of the greater expansion of blood volume and increased cardiac workload, women with twin pregnancies are more prone to PE manifestations such as hypertension and proteinuria.

In addition, the large placental volume in twin pregnancies and the higher likelihood of abnormal umbilical cord insertion (e.g., velamentous or marginal cord insertion) may result in prolonged placental hypoperfusion. Placental hypoxia promotes the release of inflammatory factors and oxygen free radicals into the maternal circulation, causing vascular endothelial injury and triggering hypertensive disorders of pregnancy (34). The risk of PE further increased to 15.68% among women with twin pregnancies complicated by IDA. In IDA, chronic systemic hypoxia, along with ischemic and hypoxic acidosis of the placental tissue, can damage capillary walls and increase vascular fragility. Furthermore, anemia leads to activation of the renin–angiotensin–aldosterone system as the kidneys sense a relative reduction in circulating blood volume (35).

The consequences of PE extend beyond pregnancy. Women with a history of PE have higher risks of cardiovascular disease, diabetes, and dyslipidemia (36). Their offspring are also at increased risk of neurodevelopmental disorders such as hyperactivity, autism, and cerebral palsy. These findings highlight the importance of early prevention, timely identification, and effective management of iron deficiency in twin pregnancies to reduce the incidence of PE and its serious maternal and fetal complications.

Women may experience a state of “physiological anemia” during pregnancy because of the disproportionate increase in plasma volume relative to RBC volume. However, the diagnostic definition of IDA in twin pregnancies remains debatable. Some studies have suggested that the optimal cutoff value for HGB is 97 g/L, as concentrations below this threshold eliminate the influence of hemodilution and better reflect the pathological state of anemia (37). Zeng et al. reported that the reference interval for HGB in healthy Chinese nonpregnant women was 115–150 g/L; based on this standard, approximately 65% of women with twin pregnancies fell outside the range, with a mean HGB of 112.6 ± 24.1 g/L in the late-pregnancy group. These findings indicate the need to establish reference intervals specific to twin pregnancies (38). Current Chinese guidelines for anemia in twin pregnancies recommend different HGB cutoffs by gestational age: <110 g/L in early pregnancy, <105 g/L in the second trimester, and <110 g/L in late pregnancy. At the same time, an SF concentration <30 μg/L supports the diagnosis (39).

A one-unit increase in log plasma ferritin was associated with a 1.20-unit increase in log Hepc intensity (40), suggesting that Hepc regulates iron balance and participates in the occurrence and progression of IDA-related disorders during pregnancy. Hepc levels are influenced by circulating and stored iron, inflammation and infection, and erythropoietic activity (41). Under conditions of iron deficiency, hypoxia, or anemia, erythropoiesis increases and Hepc expression is inhibited. When Hepc levels are low, their internalization and degradation effect on FPN is reduced, leading to enhanced intestinal iron absorption and mobilization of stored iron from macrophages and hepatocytes.

In this study, serum Hepc concentrations in women with twin pregnancies complicated by IDA were lower than those in women without IDA, which may reflect the positive feedback effect of iron levels on Hepc. Hepc expression is also physiologically suppressed during pregnancy (42). Levels decline progressively with advancing gestation and reach their lowest point in the third trimester (43), which may represent a protective mechanism to meet the body’s high iron demands. Through the iron–Hepc–FPN pathway, the body increases iron absorption and mobilizes stored iron to maintain appropriate plasma concentrations.

In this study, the occurrence of IDA in women with twin pregnancies was closely related to Hepc levels, supporting its value in IDA diagnosis. Serum Hepc has been reported to be a more effective diagnostic indicator of IDA in pregnancy than HGB, SF, or total iron-binding capacity (15). However, its diagnostic performance is optimal only when combined with HGB (44).

This study showed that low Hepc levels were a risk factor for adverse outcomes, including preterm birth, PE, placental abruption, and small-for-gestational-age neonates, in women with twin pregnancies complicated by IDA. The AUC of serum Hepc for predicting adverse maternal and fetal outcomes was 0.826, with an optimal cutoff value of 11.83 ng/mL. FPN and HGB also demonstrated good predictive performance. When HGB, Hepc, and FPN were combined, the AUC increased to 0.940, with high sensitivity and specificity. These findings indicate that Hepc, FPN, and HGB have predictive value for adverse perinatal outcomes in women with twin pregnancies complicated by IDA, and that their combined predictive efficacy is superior to that of any single marker. Therefore, in clinical practice, joint measurement of HGB, Hepc, and FPN may improve the prediction of adverse pregnancy outcomes, enabling earlier detection and treatment of IDA and its potential complications. Nevertheless, the regulatory mechanisms of Hepc during pregnancy remain unclear (45).

For twin pregnancies, it is necessary to provide guidance on weight management, nutrition, and exercise tailored to each stage of pregnancy. Especially in the late stage of pregnancy, we recommend increasing the intake of important vitamins and minerals. Pregnant women with multiple pregnancies should routinely take multivitamin tablets. If they have anemia, they should also take iron supplements (or iron-folate complex supplements).

This study focused on conducting a prospective study on twin pregnancies complicated with IDA. The levels of Hepc and FPN were detected by ELISA, and the relationship between the serum Hepc and FPN levels and IDA was analyzed. The predictive ability of each index for adverse pregnancy outcomes of IDA twins was evaluated. The results show that serum Hepc, especially the combination of FPN and HGB, has good value in predicting the adverse outcomes of IDA in twin pregnancies. The detection method of Hepc is simple and suitable for clinical promotion and application in hospitals at all levels.

This single-center study also has some limitations. Although the study hospital was a provincial tertiary referral center for the diagnosis and treatment of twin pregnancies, the number of eligible cases was relatively small, and some patients did not meet the inclusion criteria. In addition, some women were referred from primary hospitals with incomplete prenatal examination records. Difficulties in blood collection may also have introduced selection bias and systematic error. Despite controlling these indicated early delivery, larger studies could confirm the impact of IDA vs. indication for complicated twins and monoamniotic monochorionic twins. Therefore, multicenter studies with larger sample sizes are needed to further clarify the clinical utility of Hepc and FPN.

## 5 Conclusion

Twin pregnancies complicated by IDA may increase the risk of adverse perinatal outcomes such as preterm birth and PE. Early identification, prevention, and treatment of IDA are therefore critical for improving maternal and fetal prognosis. Serum Hepc is closely associated with anemia indices and has diagnostic value for IDA in twin pregnancies. Moreover, serum Hepc, particularly when combined with FPN and HGB, provides strong predictive value for adverse outcomes in this population.

## Data Availability

The original contributions presented in the study are included in the article/supplementary material, further inquiries can be directed to the corresponding authors.
